# Frying eggs or making a treatment plan? Frictions between different modes of caring in a community mental health team

**DOI:** 10.1111/1467-9566.13346

**Published:** 2021-07-11

**Authors:** Christien Muusse, Hans Kroon, Cornelis L. Mulder, Jeannette Pols

**Affiliations:** ^1^ Trimbos Institute Netherlands Institute of Mental Health and Addiction Utrecht The Netherlands; ^2^ Department Ethics, Law & Humanities Amsterdam UMC Amsterdam The Netherlands; ^3^ Tranzo, School of Social and Behavioural Sciences Tilburg University Tilburg The Netherlands; ^4^ Department of Psychiatry Erasmus MC Rotterdam The Netherlands; ^5^ Parnassia Psychiatric Institute The Hague The Netherlands; ^6^ Department of Anthropology University of Amsterdam Amsterdam The Netherlands

**Keywords:** community mental health care, continuity of care, deinstitutionalisation, modes of ordering

## Abstract

In this article, we conduct an empirical ethics approach to unravel the different perspectives on good care that are present in a community mental health team (CMHT) in Utrecht. With the deinstitutionalisation of mental health care, the importance of a close collaboration between the social and medical domains of care on the level of the local community is put in the foreground. Next to organisational thresholds or incentives, this collaboration is shaped by different notions of what good mental health care should entail. Using the concept of modes of ordering care (Moser 2005), we describe five modes of ordering mental health care that are present in the practice of the CMHT: the medical specialist, the juridical, the community, the relational and the bureaucratic perspective. These different modes of ordering care lead to frictions and misunderstandings, but are mutually enhancing at other times. Unravelling these different modes of ordering care can facilitate collaboration between professionals of different care domains and support a mutual understanding of what needs to be done. More so, the analysis foregrounds that ordering care from a relational approach is important in daily practice, but is in need of stronger legitimation.

## INTRODUCTION

How should we care for people with severe mental illness in the community? This question about how to shape deinstitutionalisation is often addressed in organisational terms. This paper addresses the question from an empirical perspective on care practices: we describe how a team of health‐care professionals in Utrecht, the Netherlands shape care in the community for people with severe mental illness (SMI)^1^ and where this leads to problems. We ask ourselves which different modes of ordering mental health care are present in the practice of the Community Mental Health Team (CMHT) and we analyse how these modes suggest different types of problems that require particular solutions in order to create good care. We show how the different modes of ordering care relate to each other – sometimes in a mutually enhancing way, but sometimes leading to friction and misunderstandings about how to proceed. By articulating these tensions, we hope to clarify what is at stake.

The study of care practices and their tensions have been approached in different ways. Navne and Svendsen (2018), Pols (2006), Brown and Korczynski (2017) and Zuiderent (2015) address the role of institutional practices, such as the process of decision‐making, the standardisation of care and accountability practices. Other work highlights the role of professional competition in the way different perspectives on care relate to each other (see, e.g., Sanders & Harrison, 2008). In this paper, we focus on notions of good care that are not attached to a specific institution or occupation. We draw on John Law’s (1994) concept of ‘modes of ordering,’ as developed for the analysis of care practices by Ingun Moser (2005). We use the concept to unravel the frictions we observed during our fieldwork. The plural ‘modes’ opens up the space to account for the fact that people, occupations or institutions can draw on different modes of ordering. As Moser (2005: 669) points out, ‘in practice people are not caught in any one mode of ordering (…) but rather slip and move between multiple modes of ordering that co‐exist, [and] are partially related in complex ways and even folded into each other’. The verb ‘ordering’ directs us to the fact that good community mental health care is not a static state or an end goal, but an ongoing process: of discussing, reframing and trying out what works. Analysing the activity of ordering helps us to render intelligible the recurrent discussions and misunderstandings in the work of the team and its partners. We analyse the work and orderings of professionals working in everyday care: the case managers, clinical nurses and others.

To answer the questions raised in this paper, we turned to the daily practice of caring, by using ethnography as the main method. For each mode of ordering care, we first describe what is seen as a problem and then link this to specific notions of good care. Analysing care situations in this way relates our analysis to the work to an *empirical ethics of care* (see, e.g., Mol et al., 2010; Pols, 2015; Willems & Pols, 2010). By describing practices and the notions of the good that are embedded in these, we follow Pols et al. (2019) who states that *‘*empirical ethics combines a “sociology of the good” (Thévenot, 2001, see also Boltanski and Thévenot 2006) and a material semiotic approach that does not ‘apply’ theoretical concepts, but studies what these concepts come to be – or how they become enacted – in specific contexts’. (Pols et al., 2019:100).

## BACKGROUND

### The context

The first author conducted fieldwork in a CMHT in Utrecht, the Netherlands. The team came into existence in 2016 due to changes in the policy and funding of Dutch mental health care that started around 2005, which prioritised the reduction of beds in psychiatric hospitals. The changes were aimed both at improving care by moving towards more community‐based and recovery‐orientated approaches and at reallocating resources and improving efficiency (Ministerie van VWS, 2012).

In Utrecht, this move towards deinstitutionalisation was the incentive to restructure care for people with so‐called SMI. Across the region, care is now decentralised into different CMHTs, in which specialised mental health treatment and supported living teams work side‐by‐side to provide care in the community for people with SMI (Taskforce EPA Midden Westelijk Utrecht, 2015). The hope is that this way of working can close the gap between mental health *support* (e.g. assistance with daily activities, the household and administration) and mental health *treatment* (evidenced‐based interventions, medication and hospitalisation), as noted in policy documents (Commissie Toekomst beschermd wonen, 2015). The other aim is making care more easily accessible by decentralising mental health care to local communities.

### The neighbourhood

The first author conducted participant observation in one CMHT located in a neighbourhood on the outskirts of Utrecht. Built in the post‐war period, the neighbourhood contains mostly high‐rise buildings with apartments that are rented out by social housing companies. It has 34,000 inhabitants from diverse cultural backgrounds, many of whom face problems like poverty, unemployment, school dropout or criminality. Furthermore, the intensity of demand for and supply of care is relatively high (Zorgverzekeraars Nederland, 2014).

### The team

The newly formed CMHT consists of care workers from two different organisations. The first is a mental health‐care organisation that provides *treatment* in different forms.^2^ Staff include a psychologist, a psychiatrist, an expert by experience, a specialised mental health nurse and mental health nurses. In their work, they adopt Flexible Assertive Community Treatment (FACT), a care model that combines individual case management with shared caseload and assertive outreach (Nugter et al., 2016; Van Vugt et al., 2011). The second constituent organisation is responsible for sheltered housing and supported living in the community.^3^ The workers involved are personal case managers and an expert by experience. Other professions, such as a rehabilitation worker and addiction care specialist, are linked to the team on a consultation basis.

The CMHT thus consists of people from two organisations, working in one team. In practice, this means that professionals in the CMHT work with different administrative structures and different accountability systems. The funding of these forms of care is also different: the supported housing organisation is funded by the social support act (Wet Maatschappelijke Ondersteuning in Dutch), which is a collective act under the responsibility of the municipality, while the clinical treatment team is paid by health insurance companies, based on individual diagnoses. This way of financing care therefore bears the risk of enlarging the observed gap between medical and social services (Mason et al., 2015). The combined CMHT tries to bridge this gap.

## METHODS AND ANALYSIS

This article is based on data collected during fieldwork carried out in a CMHT in Utrecht.^4^ The first author observed the practice of daily care in the CMHT, with a special focus on notions of good care that are at stake in these practices. To do so, she joined the team in two periods of first three and then two months, accompanying daily team meetings, house visits, and other meetings and activities at the outpatient clinic. She also paid frequent visits to sheltered housing accommodations and a local recovery college^5^. During the fieldwork, the first author observed activities and discussed these activities in interviews or more informal conversations with those involved. The fieldwork periods were separated by a break of three months, in order to analyse material and to gain a better understanding of the more long‐term developments in the team. At the end of the fieldwork, a group discussion with the team was conducted.

Alongside the participant‐observation, several standalone interviews were conducted. Eight important partners of the CMHT were selected based on the fieldwork. These informants work for respectively the police, the MH crisis department and clinic, the social welfare team, a housing association, the municipality, an association for rehabilitation and work, and a GP practice. Additional interviews were also conducted with five team members to further clarify the first author's observations and to reflect on their work. At the end of the fieldwork, six service users known to the researcher from previous house visits were approached through their case manager for an interview about their experiences with care and support from the CMHT. The selection of service users was based on previous fieldwork and the willingness of people to participate.

### Data analysis

The field notes and interviews collected during the fieldwork periods were transcribed and then coded in MAXQDA using modes of ordering as an theoretical framework. The process of coding involved open and selective stages. In the open stage, to sharpen the focus of the research and be sensitive to new questions, the material was analysed during the fieldwork periods. After the fieldwork it was then read multiple times and openly coded; parts were highlighted to identify recurring themes or patterns. This analysis was discussed with the research team and a group of qualitative researchers to strengthen the coherence of the analysis. In the selection stage, we used a second round of analysis to identify cases or situations that were indicative of different modes of ordering community mental health care.

### Ethics

Ethical approval was obtained from the research institute at the Free University of Amsterdam, and the care institute where the research was conducted.^6^ Informed consent was obtained from workers of the team, in formal interview situations and for house visits. During site visits and meetings, the researcher was always open about her role, and in the waiting area of the CMHT information about the research was provided, including a picture of the researcher and her contact details. All material was anonymised, and no names or other personal details were collected. Pseudonyms are used in this text.

## FINDINGS

In this section, we will present the analysis of the five modes of ordering we could unravel from the practices. Each mode describes a specific way of defining what the problem is, the solutions to it, and how this aligns with a specific notion of good care. To illustrate the different modes and how they relate to each other, we introduce each mode with a story about Building U, a U shaped appartement building, located in the neighbourhood where the CMHT provides its care. Many of the CMHT’s clients are living in this building.

### Mode 1: ordering care as a medical specialisation

The first time I visited Building U was together with a case manager from the CMHT. We visited a middle‐aged man called Tinus. The team has received complaints from his neighbours that he is moving furniture around at night time. He is often angry and the team thinks he is becoming more suspicious, possibly caused by an increased use of amphetamines. The CMHT wants him to start anti‐psychotic medication, but Tinus refuses, since he does not experience problems. The team is contemplating pursuing a legal decision for coercive care.

#### What is the problem?

Within the CMHT, one of the ways of defining a problem was to focus on an individual and her or his mental health diagnosis, based on the classification of symptoms. Care is ordered as a medical specialisation. In this mode, treating a person with an illness is central to providing good care. In the story about Building U, treating Tinus’ illness for instance should reduce his anxiety and thereby the nuisances he causes his neighbours. Hence, we found this problem definition as well in a discussion about another individual's eligibility for support:

The discussion in the morning meeting revolves around the fact that a man in the team’s care currently has no psychiatric symptoms. A caseworker asks: ‘But drinking because of stress is problematic, isn’t it?’ ‘It is’, confirms the psychiatrist, ‘but he is not acutely psychotic. The question then is if we should look for a solution for his problems in mental healthcare or whether social services should support him with finding a place to live’ (fieldnotes).

In the example above, the importance of psychiatric symptoms to qualify for mental health treatment is highlighted. If the problem is not medical, then a solution for the identified problems might be sought elsewhere. Ordering care from the perspective of medical specialisation thus creates a clear division between treatment and support from social services.

#### What is good care?

When mental health care is ordered as a medical specialisation, then care ideally follows a clear trajectory: a call for help from a patient, an intake to assess whether there is a psychiatric problem, a diagnosis, a treatment plan with effective interventions, and the monitoring of progress. This framework of evidenced‐based medicine is meant to optimise good care for patients and is based on the philosophical assumptions of psychiatry as a medical enterprise (Ralston, 2013).^7^ A specific aspect of this mode of ordering is the relation between care professional and patient. The professional's role is to offer the best or most effective treatment possible, based on clinical knowledge. The patient is an individual with a specific illness diagnosed on the basis of a cluster of symptoms. Care should be aimed at relieving the symptoms in the most effective way and is therefore also time‐limited: if symptoms decrease or stabilise, specialised treatment is no longer necessary.

### Mode 2: legal legitimation

Talking to the local police chief about Building U, he sees a group of inhabitants who are causing problems and are in need of support, but they will not call for help or accept support when offered. He formulates the problem in terms of a lack of a mandate to take action when people do not ask for help. ‘I would love to work more proactively, but in practice things have to escalate before something happens’.

#### What is the problem?

For the treatment offered by the CMHT to work, it requires a motivated, consenting patient. Without this, the staff faces two problems: the treatment path will stagnate, and questions about which ways of intervening are legitimate come to the fore.

A clinical nurse brings the case of Mr. Jansen to the morning meeting. He lives in a sheltered housing facility and his condition is declining daily. Clinical nurse: ‘The workers of the housing facility ask us “to do our job.” But I wonder: what exactly is my job and is what we are doing right? How far do you go if somebody neglects himself? I try to stimulate him to take his meds, I try psycho‐education, to activate him (...)’.The psychologist adds: ‘I would want to offer him CBT,^8^ but he is not motivated…’ (fieldnotes)

The above example is not an exception. In the practice of the CMHT, patients‐like Tinus whom we encountered in Building U‐ often do not explicitly ask for help or consent to the offered interventions. Then, the problem of ‘how far one can go’ comes to the fore:

In a group conversation about my research, we discuss ways of intervening, besides a juridical intervention. One of the case managers recalls that he accompanied a service user to the dentist, even drove him there. ‘It worked’, he adds, ‘the next time he went on his own’. They did, however, have a discussion about it in the team, he says, because ‘you still intervene in somebody’s life’. (CMHT group discussion).

In this example, all forms of care that intervenes in someone's life are problematised as interference with a person's autonomy. Yet this raises another crucial question: How to care without interfering? From a legal perspective, if somebody does not consent, the only option to care is to wait until the situation escalates and then intervene on legal grounds.

#### What is good care?

A specific aspect of ordering care from a legal perspective is that the patient is an autonomous individual and has the legal right to self‐determination. This autonomy is underlined by the juridical principle of informed consent. Care providers should ideally be open and transparent about different treatment options and should refrain from interfering in the process of decision‐making (Widdershoven et al., 2000). This juridical view on autonomy is closely linked to and sometimes interwoven with the ordering of care as a medical specialisation. The difference is that it is not the doctor who knows how best how to treat an illness, but rather an autonomous patient that must decide on different treatment options. In this logic, interfering in a patient's life – especially without explicit consent – is problematic. The only moment that interference becomes legitimate is when a patient is no longer seen as able to make autonomous decisions, or when he becomes a danger to himself or others. At that point, the juridical status changes and forced care is possible.^9^

### Mode 3: Caring for the local community

A safety manager of the municipality describes Building U as a cluster of people with a web of problems. In the building she sees illegal activities ranging from drug dealing to illegal prostitution. She wants to start a project with different stakeholders to address these problems on a community level.

#### What is the problem?

In this third story about Building U, it is not only the individual but also the community that is in need of care:

‘I remember situations in which I thought: this no longer holds. A situation in which you couldn’t find a place for someone. That is a difficulty, because it is key for the community approach’s success or failure. Those neighbours are looking at you. You are the health professional. And if they think: “Oh, you do not act, you just let us rot. That’s how things work?” (…) That is what I mean with trust: you have to show it, to the neighbourhood and the community. You have to show them that you want to be there for them. You are not only there for the service user, but also for the neighbours’. (Interview, worker social welfare team).

In ordering care in terms of the local community, two instruments are seen as important. The first is outreach and working proactively. Providing care is not necessarily limited to people with a formal mental health diagnosis; outreach for those not yet in care is thus a legitimate action, because it can prevent escalations that can harm the whole local community.

The second instrument is working together on the level of the local community and to share responsibility by forming alliances between care professionals and social welfare organisations:

When I enter the meeting room, professionals from the local social welfare team, the supported living team, the GP, a general practice mental health professional, a mental health nurse, and the team leader of the CMHT are present. They are here for a monthly lunch meeting to discuss ‘complicated cases’, with the idea of sharing the responsibility. At the beginning of the meeting, the GP addresses me personally: ‘It is important for your research to take on board that in here, social and medical organizations sit around the table on the level of the local neighbourhood to organise a strongly‐built basic care network. We want to learn how to do this by mutually discussing individual cases’. This morning, they discuss the difficulties of providing care for a woman with multiple problems. They first discuss how to restore contact between the woman and mental healthcare services, but as the discussion evolves and her social situation is described in more detail, they decide to start with smaller steps to decrease her social isolation. (fieldnotes).

In this mode of ordering care, there is an idea of shared responsibility to take care of the more vulnerable members of the local community. Ideally, this means shifting the perspective from one's own organisational logic towards a mutual community perspective. This shared responsibility is not always easy to coordinate among the separate organisations. Furthermore, privacy laws make it difficult to share knowledge. But different actors find innovative ways to overcome these obstacles, for instance by holding consultation meetings as the one described above.

#### What is good care?

In contrast to ordering care from a medical specialist perspective, with its focus on the individual patient, good care from the perspective of the local community includes all local citizens. It also means being accountable for people even where it is not (yet) clear if there is a diagnosable mental disorder. In contrast to the juridical mode of ordering care, intervening without consent can be the right thing to do if the wellbeing of the local community is taken into account. Continuity of care and assertive outreach are important in this mode of care. Furthermore, problems that CMHT service users face daily, such as poverty, nuisances and poor living conditions, are actively addressed with other partners.

### Mode 4: A relational approach

Speaking to a social worker about Building U, he stresses that care is not only about taking action, but also about working on relations: ‘People have to get to know you and you have to know them. It is about trust (…). You need caregivers who, so to speak, crawl into the building’.

#### What is the problem?

In this mode of ordering care, the problem is not so much the illness itself but people's relations and networks. Relating – especially initiating and maintaining contact – is seen as an important part of the work of the CMHT, especially if people are avoiding care. The team has developed creative ways to establish relations:

In an interview with a mental health nurse, we discuss the scope of her work and the importance of patients being motivated for treatment. Then she makes a switch: she refers to a situation in which she does not follow her strict task description as a mental health specialist:

R: (…) ‘I have a client’, she recalls, ‘with him, trust is really difficult and his house is a mess. He is 23 years old, dependent on alcohol and cannabis, and looks really bad. Then I come in and I fry him some eggs. (…)Those are the good parts of the job, the flexibility’.

I: ‘So on the one hand there should be a request for help and a treatment plan…’

R: ‘Yes, but thinking outside the box is also very important’ (Interview, clinical nurse).

The clinical nurse shifts from ordering care as a medical specialisation to ordering care from a relational approach, by mentioning that in some situations she combines the role of being a nurse with frying eggs. Theoretically, this could lead to friction about which action is right, yet in practice she cherishes this double‐sidedness. What is ‘good’ here is being flexible, thinking outside the box, doing what is necessary to make contact and motivate others for certain behaviours.

During the fieldwork, there were many examples of professionals relating to clients in creative ways. Seduction, or what Driessen (2017) calls ‘will work’ – work aimed at aligning the other's wants with one's own – can be a part of this:

We are both working on a laptop in the ‘office garden’ of the community mental healthcare team when the case manager recalls the case of a woman who was known to be ‘difficult’; refusing care:

R: ‘So I thought of an intervention to enter the house. I knew her coffee machine was broken and I had a spare one. I rang the bell and the woman opens the door. ‘You can go ‘pierewaaaien,’^10^ the woman said. ‘Oh, you are lucky, I know the meaning of ‘pierewaaiden,’ I replied. ‘It means I have to leave, right? I will not leave. Maybe I can do some groceries for you? And here you have a coffee machine.’ Then I was allowed in and after a while she said: ‘It appears that you can stay…’ (fieldnotes)

#### What is good care?

In ordering care from a relational approach, the focus is not only on the individual, be it a citizen or a patient, but also on the relations between patients, caregivers and others. Caring is working *on* these relations – trying to establish or maintain them – and *with* these relations – to avoid a crisis, for instance (Muusse et al., 2020). Bringing in the relations between clients and caregivers is also central in an ethics of care that focuses on the interdependence of people (Tronto, 1993; Voskes, 2014). In contrast to the ordering of care as a medical specialisation, care here is not so much an outcome of a time‐limited treatment trajectory, but an ongoing process. As Pols (2006) points out, there is no clear directive or general method that prescribes how these relationships should be crafted; it depends on circumstances, personal styles and who is involved. What is the good thing to do can differ along the way.

In this mode of ordering care, it is impossible for care workers to only address mental illness as a discrete medical domain; they have to engage in non‐medical domains of peoples’ life (work, finances, health). In doing this, the division between treatment and support becomes blurred. Good care is now about doing both: frying eggs while attending to psychiatric problems.

Ordering care from a relational perspective offers the legitimation for care professionals to intervene in non‐medical domains of peoples’ lives. This is what the social worker refers to in his story about Building U, when he talks about the need for people who are able ‘to crawl into the building’, to make contact with the inhabitants of Building U. Good care is building trust, knowing people and intervening when necessary from within the established relationship.

### Mode 5: Bureaucratic accountability

To address the problems in Building U the worker of the social welfare team points out that the way care organised should support the relational approach: ‘You need people who have the time to work without a fixed caseload, to reach out to the people who are not seen by anybody’.

#### What is the problem?

In this mode of ordering care, the organisation and finical structure defines what is good care. In this mode, two problems are central: rising health‐care costs and waiting lists. To solve these problems, care should be efficient and cost effective. A way to achieve this is by making the organisation and accountability of care more transparent and regulating the working time of professionals.

We specify what this means for clinical nurses in the team, because observations showed that they most frequently juggled with the accountability system they had to work with.^11^ To receive treatment from the CMHT, you need to have a diagnosis so that a DBC can be opened. DBC stands for ‘diagnose behandel combinatie’ and is a registration system in which clinical workers have to register all their working hours in order to get their work reimbursed by the health insurance company. This is a quite specific task. Richard, a specialised clinical nurse, shows me how it works:

He shows me a computer screen that consists of four parts. Part one is about productivity: Richard’s exact working hours are set against the norm set by the institute. Every moment of the day should have a code. I ask whether these norms influence his work. ‘Not directly’, he replies, ‘our team leader is understanding. But the system sometimes irritates me. I try to keep thinking: I do what I have to do for the patient’. (fieldnotes)

Here Richard frames the tension the system is causing him as an ethical dilemma: he feels stress when he is not living up to the norms set by the system, but he feels ethical commitment to do what ‘he has to do for the patient’. Similar dilemmas are described by Brown and Korczynski (2017) in studying home care work. They describe how by spending more (private) time with patients than provided by the system, care workers found a way to keep their autonomy and their ideals of what good care entails (the ‘caring self’). We observed a similar strategy: clinical workers offered the care they perceived as necessary and then tried to fit it in the accountability system as much as possible. This does not mean that how care is legitimised does not influence their practice of caring. The protocols for opening a DBC for instance play an important role in the decision‐making process of who qualifies as a patient for the team.

#### What is good care?

One of the goals of the DBC system is to increase transparency regarding price and performance (Tummers, 2010), thus making care more transparent and efficient. This specific way of financing and organising mental health care and the corresponding accountability system is normative; it strengthens the idea that care should be focused on an individual with diagnosable, treatable problems. It therefore supports the ordering of care from a medical specialist perspective, while creating problems to account for other forms of care that are not directly linked to caseload reasoning and temporality, with a clear diagnosis and linear treatment path. This encompasses activities such as caring for the local community or a relational approach, which cannot be paid for using this DBC system.

## MODES OF ORDERING THAT CLASH OR ENHANCE EACH OTHER

As can be seen in the examples above, the different modes of ordering care sometimes enhance each other, but at other moments they lead to frictions. How does this work? We bring in one more example:

The discussion starts with a remark by the psychologist about how far the team’s responsibility reaches: ‘What is our responsibility if somebody is referred to our team with a stack of problems, maybe for over 20 years already?’

The psychologist remarks that the team sometimes takes people into care even when they lack the specific expertise, because the person is not receiving treatment from other diagnosis‐specific outpatient teams that do not engage in outreach. She points out that one of the differences is that the CMHT, in contrast to those teams, works on demand. ‘You could say that we are then causing under‐treatment by taking somebody into care. For instance with borderline [personality disorder]. I know schema therapy is the first treatment advised, but we are not trained in it’. What the team then does is to engage in making contact, and support the patient in daily life, motivating them for care. But is this good care, or under‐treatment? The opinions differ.

A case manager comments: ‘When I started working here, I was told that this team would end the endless discussions about which patient belongs where. But in practice it is not solved at all’. A clinical nurse adds: ‘When I started, I had the assignment to get people into treatment who are hard to engage, to work on outreach. But in our team there is a strong focus on the diagnosis and if it is treatable, yes or no. If not, then we refer somebody to a social worker or the GP’. She recalls the case of Anna, a woman with a long care history, dealing with trauma, personality disorder and addiction problems, but who lacked treatment motivation. ‘If we then state: we have to refer her because we are not really treating her, I do not think that is correct. In fact, we do a lot: we make contact, we stabilise the situation. She is doing better, despite the persisting problems’.

Second nurse: ‘I want to add something positive. We have a lot of these kind of referrals, so obviously people can find us and that is because we are now [known] in the local neighbourhood’ (Fieldnotes).

In the above discussion, different modes of ordering care come to the fore. First, the psychologist problematises taking people into care who are not getting appropriate care, based on their diagnosis. Can you consider this good care, or is the team facilitating under‐treatment? This question reflects the mode of ordering care as a medical specialisation, in which good care is connected to a diagnosis and evidenced‐based interventions. Second, the clinical nurse points out the team's over‐concentration on diagnosis and that supporting people in daily life is just as important. Here, care is ordered from a relational approach; it is about different life domains and building relations. Third, there is the perspective of the local community, which is brought in by the final remark from the second nurse. The fact that a lot of people who have problems obtaining care elsewhere end up in their team's support is not seen as a problem, but as something positive: obviously they are being successful in making connections with the local community.

In the example above, the modes of ordering mental health care as a medical specialisation and a relational approach clash. From the perspective of relational working, care can be more than clinically proven treatment: supporting daily life is also part of good care and is seen as the task of the CMHT. This is especially so for people facing multiple problems, but who are hard to engage in care or where there is no clear treatment request. For this group, it is much harder to set out a clear time‐limited treatment path, aimed at symptom relief.

The tensions between the two modes do not arise on a level playfield. Ordering care as a medical specialisation is supported by the idea of evidenced‐based working and the DBC bureaucratic way of ordering care, while ordering care from a relational perspective has no such strongly articulated legitimation and a lack of professional specialisation. This distinction is strengthened by the policy decision to finance treatment and support separately. This lack of legitimation makes a lot of the daily relational work of the CMHT’s clinical nurses and case managers precarious; nevertheless, the need to work in this way is often clear to all involved.

The role of these institutional dynamics in legitimising care resonates with Lester’s (2009) analysis of the way clinicians in an eating disorder clinic must make translations between different care frames. In this process, clinicians have a role she describes as ‘brokers’ wherein they reframe their own descriptions of what is going on in terms of the managed care model used by insurance companies. Lester describes how in these everyday ethical negotiations clinical workers need to balance contradictory or conflicting imperatives to legitimise their actions and decisions.

This ethical distress that institutional strains put on caregivers to legitimise specific ways of caring is also highlighted by Brodwin (2013) who points out that the everyday ethical questions clinicians in a community mental health team have to face are not only about what constitutes as good mental health care, but also who will benefit the most from treatment. He describes how clinicians thus become involved in forms of ‘boundary work,’ (Gieryn, 1983). In doing so, clinical workers take on the role of gatekeepers to care. In our team, this boundary work got shape by questioning the legitimacy of the treatment of people who do not fit easily in the medial specialist mode of ordering care, such as Anna in the example.

It is important to note that these discussions are taking place against the background of the CMHT’s position in the local care landscape, which contains highly specialised polyclinic teams organised around a specific diagnosis, and the CMHT, which also offers specialised mental health care, but is not aimed at a specific diagnosis and is embedded in the local community. So the CMHT has a double assignment: its mission is to be both present and engaged in the local community *and* to provide highly specialised mental health care. The team must care for all and care for a few at the same time.

Ordering care from a medical specialist perspective versus a relational perspective is not, however, in tension in all situations: if a relational approach is lacking, then the team knows that in practice there is the risk that treatment will never reach the patient or will be less successful. A relational approach is thus no longer in tension with, but a prerequisite or partner for, specialised care. The workers of the CMHT know this from experience, and fry eggs while making up treatment plans.

## CONCLUSION AND DISCUSSION

In this paper, we described dilemma's related to multiple perspectives on good community mental health care, using multiple stories about Building U. We unravelled the stories as different modes of ordering care that are present in the daily discussions about the work of the CMHT. Below, we highlight three important contrasts between the different modes of ordering care:

First, in the different modes of ordering care, the objects of care differ. In ordering care as a medical specialisation, the object of care is an individual with an illness. In contrast, in the community and relational approach, care is not only aimed at the individual but also the local community; it takes into account the fact that people are part of a social network. These forms of collective care fit poorly, however, into the way in which care is organised and financed around caseloads. This tension is all the more clear in the double task of the CMHT, in which workers simultaneously have to care for a few (those in need of specialised care) and for all (the local community). Combining these two tasks puts a strain on the CMHT workers, especially because work that is not registered as ‘productive’ in the DBC’s terms is much more difficult to justify.

Second, the different modes of ordering care emphasise different views of autonomy. The juridical and medical specialist modes entail an individualised, negative concept of autonomy or freedom, namely freedom from interference (Berlin, 1969; Widdershoven et al., 2000). In contrast, in the community perspective or relational approach, the emphasis is not so much on the individual but on the relations between people and things. In these modes of ordering care autonomy is viewed relationally: from within relations, options for interference, outreach and will work are explored. We observed these clashing visions of autonomy in discussions about the legitimacy to interfere in a person's life. Unravelling the different modes of ordering at stake in these discussions can help to sharpen the formulation of these dilemmas and open up creative ways of dealing with the binary opposition between coercion and care that is often created (Jerak‐Zuiderent, 2012; Lawn et al., 2016).

Third, the different modes of ordering care entail different ideas about continuity of care. Is care a temporary intervention or a long‐term, more cyclical process (Hautamäki, 2018; Lester, 2009)? This question was important in the discussions about whom to take in as a patient and whom to refer elsewhere. In a relational mode of ordering care, building relations and trust is essential and takes time. But the caseload approach to ordering care structures care as a more temporary intervention. This opposition between temporality and continuity of care at moments causes insecurity about what is proper care or treatment.

### What can we learn from this?

Different notions on good care are often described and explained from an organisational perspective or in the light of professional competition. In our research, we approached the question by turning to daily care practices instead, with ethnography as our main method. In our analysis, we followed Thévenot (2001) by describing the ‘variation (..) of what is good’ (ibid:59) in care practice. The concept of modes of ordering points to the way these goods, and also what is taken to be the problem is part of a particular way of ordering practice (Moser, 2005). With this approach, our research demonstrates that people are not necessarily caught up in one mode of ordering care, but shift between different modes.

Our research made visible how clinical workers in daily care have to juggle with these different notions of good community mental health care and the tensions that may arise between them. But this is not a level playing field. The medical specialist mode is strengthened by the juridical and bureaucratic perspectives, while strong institutional legitimation is often lacking for the relational perspective. At moments, this leads to insecurity among team members regarding whether the relational approach that they often engage in is indeed ‘good care’.

These findings point to the necessity for a consciousness that working in the context of these different modes creates ambivalence and other difficulties, especially in caring for those with more complex problems that are not easily addressed within a ‘managed care model’ in which care is standardised and time limited (e.g. Lester, 2009). Unravelling these different modes of ordering care can facilitate collaboration between professionals of different care domains and support a mutual understanding of what needs to be done. More so, it can give a ‘reflexive backup’ (Pols, 2006: 426) to those forms of care in lack of an administrative or organisational legitimation. The awareness among practitioners and policymakers of these dynamics could be helpful by developing new ways of facilitating (mental) health‐care workers in working together in both serving individuals with complex mental problems and the community they live in.

## AUTHOR CONTRIBUTION

**Christien Muusse:** Conceptualization (equal); Formal analysis (lead); Funding acquisition (supporting); Investigation (lead); Project administration (lead); Writing‐original draft (lead); Writing‐review & editing (lead). **Hans Kroon:** Conceptualization (supporting); Formal analysis (supporting); Funding acquisition (equal); Investigation (supporting); Supervision (equal); Writing‐original draft (supporting); Writing‐review & editing (supporting). **Cornelis L. Mulder:** Conceptualization (equal); Funding acquisition (equal); Supervision (equal); Writing‐original draft (supporting); Writing‐review & editing (supporting). **Jeannette Pols:** Conceptualization (equal); Formal analysis (supporting); Funding acquisition (equal); Supervision (lead); Writing‐original draft (supporting); Writing‐review & editing (supporting).

## ENDNOTES

^1^
SMI refers to refer to long‐term psychiatric disorders that interrupt, destabilise and imperil lives over a long period of time (Drake, Green, Mueser & Goldman, 2003). This CMHT was not diagnosis specific. Substance abuse was not an exclusion criterium.

^2^
This organization also runs the psychiatric hospital in the city centre, a crisis team and different specialised teams for specific diagnoses. The organization has several integrated CMHTs localised in specific areas providing care for people with SMI.

^3^
This organization has different sheltered housing locations in the city and provides comprehensive support to people who live independently but require additional assistance due to SMI. Some, but not all, also receive treatment from the mental health department or have done so in the past.

^4^
We studied the practice of community mental health care in one CMHT in Utrecht, as part of a larger project that contrasts community mental healthcare practices in both Trieste, Italy and Utrecht, the Netherlands, to learn more about different ways to deal with a psychiatric crisis in community care.

^5^
A recovery college supports people living with mental health problems through adult education rather than through treatment (Newman‐Taylor et al., 2016). It is based on self‐help and mutual peer support.

^6^
The medical ethics committee of the Vrije Universiteit Amsterdam (METC Vumc, FWA00017598) declared that the Medical Research Involving Human Subjects Act (WMO) does not apply to the study.

^7^
Evidenced‐based medicine relies on classification models to conduct clinical trials at the group level. The resulting information is used to improve the effectiveness of clinical practice for individual patients (van Os et al., 2019).

^8^
CBT stands for Cognitive Behavioural Therapy.

^9^
After fieldwork was completed, a new law concerning compulsory care was introduced in the Netherlands (see Frederiks, 2018).

^10^
The Dutch ‘pierenwaaien’ refers to going out, or partying, but is used in this context to tell someone to go away.

^11^
As mentioned above, part of the team is paid by the social welfare act, and accountability for their work is organised differently.

## References

[shil13346-bib-0001] Advies Commissie Toekomst beschermd wonen . (2015). Van beschermd wonen naar een beschermd thuis, p. 52. Available at: https://vng.nl/files/vng/van‐beschermd‐wonen_20151109.pdf

[shil13346-bib-0002] Berlin, I. (1969). Four essays on liberty. Oxford University Press.

[shil13346-bib-0003] Brodwin, P. (2013). Everyday ethics. University of California Press.

[shil13346-bib-0004] Brown, K., & Korczynski, M. (2017). The caring self within a context of increasing rationalisation: the enduring importance of clients for home care aides. Sociology, 51(4), 833–849. 10.1177/0038038515608112.

[shil13346-bib-0005] Drake, R. E., Green, A. I., Mueser, K. T., & Goldman, H. (2003). The history of community mental health treatment and rehabilitation for persons with severe mental illness. Community Mental Health Journal, 39, 427–440. 14635985 10.1023/a:1025860919277

[shil13346-bib-0006] Driessen, A. (2017). Sociomaterial will‐work: Aligning daily wanting in Dutch dementia care. In J. Boldt, & F. Krauze (Eds.), Care in healthcare: reflections on theory and Practice (pp. 111–133). Palgrave Macmillan. 31314350

[shil13346-bib-0007] Frederiks, B. (2018). New Dutch Care and Coercion Act: consequences for caregivers and clients. Journal of Applied Research in Intellectual Disabilities, 31(4), 583.

[shil13346-bib-0008] Gieryn, T. (1983). Boundary‐work and the demarcation of science from non‐science: strains and interests in professional ideologies of scientists. American Sociological Review, 48(6), 781–795. 10.2307/2095325.

[shil13346-bib-0009] Hautamäki, L. (2018). Uncertainty work and temporality in psychiatry: how clinicians and patients experience and manage risk in practice? Health, Risk and Society, 20(1–2), 43–62. 10.1080/13698575.2018.1442918.

[shil13346-bib-0010] Jerak‐Zuiderent, S. (2012). Certain uncertainties: Modes of patient safety in healthcare. Social Studies of Science, 42(5), 732–752. 10.1177/0306312712448122. 23189612

[shil13346-bib-0011] Jerak‐Zuiderent, S. (2015). Accountability from somewhere and for someone: relating with care. Science as Culture, 24(4), 412–435. 10.1080/09505431.2015.1050368.

[shil13346-bib-0012] Law, J. (1994). Organizing modernity. Blackwell.

[shil13346-bib-0013] Lawn, S., Delany, T., Pulvirenti, M., Smith, A., & McMillan, J. (2016). Examining the use of metaphors to understand the experience of community treatment orders for patients and mental health workers. BMC Psychiatry, 16(1), 1–16. 10.1186/s12888-016-0791-z. 27030136 PMC4815077

[shil13346-bib-0014] Lester, R. J. (2009). Brokering authenticity: Borderline Personality Disorder and the ethics of care in an American eating disorder clinic. Current Anthropology, 50(3), 281–302. 10.1086/598782. 19827330

[shil13346-bib-0015] Mason, A., Goddard, M., Weatherly, H., & Chalkley, M. (2015). Integrating funds for health and social care: An evidence review. Journal of Health Services Research and Policy, 20(3), 177–188. 10.1177/1355819614566832. 25595287 PMC4469543

[shil13346-bib-0016] Ministerie van VWS . (2012). Bestuurlijk akkoord toekomst GGZ 2013 – 2014. Den Haag.

[shil13346-bib-0017] Mol, A., Moser, I., & Pols, J. (2010). Care: Putting practice into theory. In: A. Mol, I. Moser, & J. Pols (Eds.), Care in practice: on tinkering in clinics, homes and farms (pp. 7–25). Transcript Verlag.

[shil13346-bib-0018] Moser, I. (2005). On becoming disabled and articulating alternatives. The multiple modes of ordering disability and their interferences. Cultural Studies, 19(6), 667–700. 10.1080/09502380500365648.

[shil13346-bib-0019] Muusse, C., Kroon, H., Mulder, C. L., & Pols, J. (2020). Working on and with relationships: relational work and spatial understandings of good care in community mental healthcare in trieste. Culture, Medicine and Psychiatry, 44, 544–564. 10.1007/s11013-020-09672-8. PMC7497456 32246246

[shil13346-bib-0020] Navne, L. E., & Svendsen, M. N. (2018). Careography: Staff experiences of navigating decisions in neonatology in Denmark. Medical Anthropology: Cross Cultural Studies in Health and Illness, 37(3), 253–266. 10.1080/01459740.2017.1313841. 28375646

[shil13346-bib-0021] Newman‐Taylor, K., Stone, N., Valentine, P., Sault, K., & Hooks, Z. (2016). The Recovery College: A unique service approach and qualitative evaluation. Psychiatric Rehabilitation Journal, 39(2), 187–190. 10.1037/prj0000179. 27148854

[shil13346-bib-0022] Nugter, A., Engelsbel, F., Bähler, M., Keet, R. V., & Veldhuizen, R. (2016). Outcomes of FLEXIBLE Assertive Community Treatment (FACT) implementation: a prospective real life study. Community Mental Health Journal, 52(8), 898–907. 10.1007/s10597-015-9831-2. 25648552 PMC5108818

[shil13346-bib-0023] Pols, J. (2006). Accounting and washing good care in long‐term psychiatry. Science, Technology, & Human Values, 31(4), 409–430. 10.1177/0162243906287544.

[shil13346-bib-0024] Pols, J. (2015). Towards an empirical ethics in care: relations with technologies in health care. Medicine, Health Care and Philosophy, 18(1), 81–90. 10.1007/s11019-014-9582-9. 25023945

[shil13346-bib-0025] Pols, J., Willems, D., & Aanestad, M. (2019). Making sense with numbers. Unravelling ethico‐psychological subjects in practices of self‐quantification. Sociology of Health and Illness, 41(1), 98–115. 10.1111/1467-9566.12894. 31599983 PMC6856866

[shil13346-bib-0026] Ralston, A. S. G. (2013). The philosophies of psychiatry: Empirical perspectives. Medicine, Health Care and Philosophy, 16(3), 399–406. 10.1007/s11019-012-9420-x. 22752587

[shil13346-bib-0027] Sanders, T., & Harrison, S. (2008). Professional legitimacy claims in the multidisciplinary workplace: The case of heart failure care. Sociology of Health and Illness, 30(2), 289–308. 10.1111/j.1467-9566.2007.01052.x. 18290937

[shil13346-bib-0028] Taskforce EPA Midden Westelijk Utrecht (2015). Volwaardig burgerschap en psychiatrie, p. 2015.

[shil13346-bib-0029] Thévenot, L. (2001). Pragmatic regimes governing the engagement with the world. In T. R. Schatzki, K. Knorr‐Cetina, & E. von Savigny (Eds.), The practice turn in contemporary theory (pp 56–73). Routledge.

[shil13346-bib-0030] Tronto, J. C. (1993). Moral boundaries: a political argument for an ethic of care. Routledge.

[shil13346-bib-0031] Tummers, L. (2010). De bereidheid van GGZ‐zorgprofessionals om te werken met DBC’s. Erasmus University.

[shil13346-bib-0032] Van Os, J., Guloksuz, S., Vijn, T. W., Hafkenscheid, A., & Delespaul, P. (2019). The evidence‐based group‐level symptom‐reduction model as the organizing principle for mental health care: time for change? World Psychiatry, 18(1), 88–96. 10.1002/wps.20609. 30600612 PMC6313681

[shil13346-bib-0033] Van Vugt, M. D., Kroon, H., Delespaul, P. A. E. G., Dreef, F. G., Nugter, A., Roosenschoon, B.‐J., van Weeghel, J., Zoeteman, J. B., & Mulder, C. L. (2011). Assertive community treatment in the Netherlands: outcome and model fidelity. Canadian Journal of Psychiatry, 56(3), 154–160. 10.1177/070674371105600305. 21443822

[shil13346-bib-0034] Voskes, Y. (2014). No effect without ethics: Reduction of seclusion in psychiatry from a care ethics perspective. 10.1177/0969733013493217 24036666

[shil13346-bib-0035] Widdershoven, G., Berghmans, R., & Molewijk, A. (2000). Autonomie in de psychiatrie [Autonomy in psychiatry]. Tijdschrift Voor Psychiatrie, 42(6), 389–398.

[shil13346-bib-0036] Willems, D., & Pols, J. (2010). Goodness! The empirical turn in health care ethics. Medische Antropologie, 22(1), 161–170.

[shil13346-bib-0037] Zorgverzekeraars Nederland . (2014). Rapportage EPA‐vignettenstudie. Zeist.

